# Spatial and temporal variability of the *Glossina palpalis palpalis *population in the Mbini focus (Equatorial Guinea)

**DOI:** 10.1186/1476-072X-6-36

**Published:** 2007-08-30

**Authors:** Jorge Cano, Miguel Angel Descalzo, Nicolas Ndong-Mabale, Pedro Ndongo-Asumu, Leonardo Bobuakasi, Jesús N Buatiché, Sisinio Nzambo-Ondo, Melchor Ondo-Esono, Agustin Benito, Jesus Roche

**Affiliations:** 1 National Centre of Tropical Medicine, Instituto de Salud Carlos III, C/Sinesio Delgado 6, 28029, Madrid, Spain; 2 National Centre of Endemic Control, Instituto de Salud Carlos III, Bata, Equatorial Guinea, Africa; 3 National Sleeping Sickness Control Programme, Ministry of Health and Social Welfare, Bata, Equatorial Guinea, Africa

## Abstract

**Background:**

Human African Trypanosomiasis is a vector-borne parasitic disease. The geographical distribution of the disease is linked to the spatial distribution of the tsetse fly. As part of a control campaign using traps, the spatial and temporal variability is analysed of the glossina populations present in the Mbini sleeping sickness foci (Equatorial Guinea).

**Results:**

A significant drop in the annual mean of the *G. p. palpalis *apparent density was noted from 2004 to 2005, although seasonal differences were not observed. The apparent density (AD) of *G. p. palpalis *varies significantly from one biotope to another. The fish dryers turned out to be zones with the greatest vector density, although the AD of *G. p. palpalis *fell significantly in all locations from 2004 to 2005.

**Conclusion:**

Despite the tsetse fly density being relatively low in fish dryers and jetties, the population working in those zones would be more exposed to infection. The mono-pyramidal traps in the Mbini focus have been proven to be a useful tool to control *G. p. palpalis*, even though the activity on the banks of the River Wele needs to be intensified. The application of spatial analysis techniques and geographical information systems are very useful tools to discriminate zones with high and low apparent density of *G. p. palpalis*, probably associated with different potential risk of sleeping sickness transmission.

## Background

Over the last 20 years, great leaps forward have been made in the study of geographical or spatial phenomena. Improvements to global positioning systems (GPS), the development of new and more powerful remote sensors, better image processing software and new geographical information systems (GIS) are some of the main advances in the study of spatial phenomena. However, the application of this technology to the study of infectious disease epidemiology is relative new and is still in its earliest stages [1,2].

Given that the spatial distribution of vector-borne diseases is demarcated by the geographical distribution of the vectors and their vertebrate hosts, the control programmes and epidemiological surveillance should include the spatial analysis of the different transmission parameters [3].

Therefore, knowledge of the spatial-temporal variability in vector abundance and distribution is fundamental to guaranteeing rational management of the vector control strategies [4,5].

Progress in the spatial analysis of point patterns, fundamentally driven by the development of geo-statistical models, has led to the spatial distribution of vector populations, principally insects, being quantified and modelled [4,6,7]. However, the spatial distribution of vectors does not always tally with the differences in the intensity of the transmission. In the case of malaria, some authors have established that the density of adult mosquitoes does not always correlate with the transmission intensity and incidence of severe malaria [8]. Other variables need to be included in the model so that the spatial and temporal differences in the transmission pattern of this type of disease can be better explained. Over recent years, risk maps have emerged that include data relating to the disease epidemiology, environmental and climatology information in the vector distribution spatial models [2].

Human African Trypanosomiasis (HAT), also known as sleeping sickness, is a vector-borne parasitic disease. The parasites are transmitted to humans by the tsetse fly (Diptera: Glossinidae), which is found in Sub-Saharan Africa. The geographical distribution of the disease is linked to the spatial distribution of the tsetse fly, even though transmission does not only occur by the presence of the vector.

Great emphasis has been placed on identifying human, environmental and epidemiological (parasitological and entomological) factors that would be related to the spatial distribution of the disease [9-11]. Geographical information systems, combined with information provided by remote sensors, have enabled environmental factors associated to the distribution of the tsetse fly populations and trypanosomiasis to be identified [9,12,13]. During a study conducted in Uganda using Landsat ETM images, it was concluded that the presence of cases of trypanosomiasis was associated with proximity to swampy zones and a sparsely populated areas [9].

In the same way, human displacements, whether voluntary (economic reasons) or forced (wars and conflicts), become risk variables when the population moves into the habitat of the vector [13-15].

The tsetse fly density and the infection rate are the main entomological factors related to the transmission risk [16]. Based on this, some authors consider that distribution vector prediction models can be used to identify and demarcate transmission risk areas. The distribution and density of potential hosts (human or animals) and land cover were taken into account when generating those models [17].

In Equatorial Guinea, four trypanosomiasis foci are described: an insular focus, which is being eliminated [18] and 3 continental foci [19], active but with a low case incidence [20]. In the four foci, *Glossina palpalis palpalis *has been identified as the main vector of the disease [15]. The initial phase of vector control was carried out in the Luba insular focus in 1985. These studies determined the distribution and density of *G. p. palpalis *and assessed the effectiveness of the mono-pyramidal traps as a control strategy [15]. In 2002, a vector control programme was implemented based on the use of traps in the Kogo and Mbini continental foci. At the begining of control campaign, global apparent density (AD) in Mbini focus was 4.19 tsetse flies/trap/day.

The objectives of this study are to analyse the spatial and temporal variability of the glossina populations present in the Mbini foci during 2004 and 2005, and identify the zones with higher apparent density of tsetse flies. Furthermore, the impact of control activities is assessed.

## Results

Between July 2004 and December 2005, a total of 7,361 *glossinae *was caught; 5,470 (74.3%) *Glossina palpalis palpalis*, 1,606 (21.8%) *Glossina tabaniformis *and 285 (3.9%) *Glossina caliginea*. During that period, a monthly average of 65 traps was operative, while the remainder had broken or had fallen at the time of being checked. A greater number of disappeared, broken and fallen traps were recorded during the rainy season, fundamentally in villages where greater resistance was noted among the population towards the control activities.

**Figure 1:**
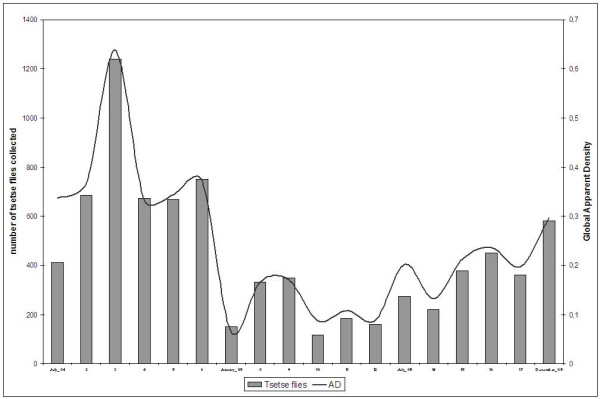
Evolution of the total catches and the apparent density (AD) in the Mbini focus (2004–2005).

The global AD in July 2004 was 0.33 tsetse/trap/day, reached a minimum of 0.06 in January 2005 and was then progressively increased until it hit 0.29 in December (Figure 1).

Table 1 sets out the mean AD (MAD) data for the three species by season and year. The MAD for *G. p. palpalis *in the Mbini focus during the 2004 dry season was 0.39 (sd: 0.39), which fell to 0.31 (sd: 0.26) during the rainy season (Mann-Whitney test, p = 0.473). The following year, the MAD was 0.17 (sd: 0.24) in the dry and 0.21 (sd: 0.26) in the rainy season (p = 0.09). A significant drop in the annual MAD was noted, from 0.35 (sd: 0.33) in 2004 to 0.19 (sd: 0.25) in 2005, for this species, despite the recovery recorded during the last quarter of 2005.

**Table 1:** Mean apparent density for the tsetse fly species caught in the Mbini focus

		***Glossina p. palpalis***		***Glossina tabaniformis***		***Glossina caliginea***	
		
		**Mean**	**± sd**^#^	**Mean**	**± sd**	**Mean**	**± sd**
**2004**	**Dry season**	0,39	0,39	0,07	0,09	0,01	0,03
	**Rainy season**	0,31	0,26	0,19	0,26	0,02	0,03
	**Annual**	0,35	0,33	0,13	0,20	0,01	0,03

**2005**	**Dry season**	0,17	0,24	0,02	0,04	0,01	0,02
	**Rainy season**	0,21	0,26	0,01	0,04	0,01	0,02
	**Annual**	0,19	0,25	0,01	0,04	0,01	0,02

^#^sd -standard deviation

A significant drop in the annual MAD was noted for *G. tabaniformis*, but not between seasons for the second year of activity (p = 0.664). In the case of *G. caliginea*, the MAD values were very low in the two-year study period, with no seasonal or annual differences being noted.

**Figure 2:**
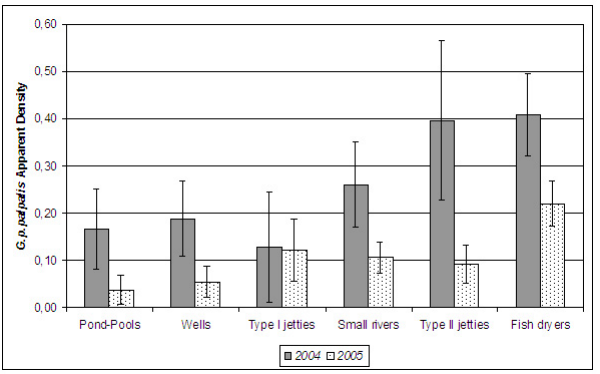
Annual mean apparent density for *Glossina p. palpalis *according to the location (habitat).

The MAD for *G. p. palpalis *varies significantly from one zone to another (Kruskal- Wallis test, p < 0.05). When analysing the differences between locations, it notes that the annual MAD for *G. p. palpalis *in 2004 was significantly higher in the fish dryers (MAD: 0.41, sd: 0.38) than in wells, ponds-pools, type I jetties and small rivers. There were no significant differences with respect to the type II jetties. In that year, the zones with lower MAD were the type I jetties, with a MAD equal to 0.13 (sd: 0.26) (Figure 2).

After a year of using mono-pyramidal traps, the differences between locations changed. The fish dryers continued to be points with the highest AD for sleeping sickness vector (MAD: 0.22, sd: 0.29), even though no significant differences were noted with respect to the type I jetties (p = 0.111). The pond-pools became the zones with the lowest AD, with an MAD equal to 0.04 (sd: 0.13).

The AD of *G. p. palpalis *fell significantly from 2004 to 2005 in all locations except for the type I jetties (p = 0.504) (Figure 2).

Table 2 sets out the total catches and seasonal MAD of *G. p. palpalis *according to the location and season. Significant seasonal differences were only noted in tributaries (small rivers) during the second control year.

**Table 2:** Total catches and seasonal MAD of G. p. palpalis according to the location and season

	**2004**						**2005**					
	**dry season**			**rainy season**			**dry season**			**rainy season**		
	
	**N**	**MAD**^#^	**± sd**^&^	**N**	**MAD**	**± sd**	**N**	**MAD**	**± sd**	**N**	**MAD**	**± sd**
**Pond-Pools**	87	0.18	0.24	86	0.15	0.29	5	0.00	0.02	68	0.07	0.18
**Wells**	65	0.10	0.14	221	0.27	0.39	67	0.05	0.18	97	0.06	0.17
**Type I jetties**	14	0.07	0.14	56	0.17	0.32	62	0.15	0.20	47	0.10	0.16
**Small rivers**	584	0.28	0.70	580	0.24	0.48	*598	**0.13**	**0.37**	*369	**0.08**	**0.19**
**Type II jetties**	234	0.42	0.69	274	0.37	0.54	108	0.08	0.21	129	0.10	0.17
**Fish dryers**	479	0.48	0.45	360	0.34	0.30	417	0.22	0.30	463	0.22	0.28

**Total**	**1463**			**1577**			**1257**			**1173**		

^#^MAD-Mean Apparent Density;^&^sd -standard deviation *p < 0.05

Table 3 sets out the quality of the best simulations performed of the prediction surfaces of the annual and seasonal AD for *G. p. palpalis *and the main descriptive parameters of each model.

**Table 3:** Statistic parameters and adjustment quality of the prediction models generated using annual and seasonal AD

		**2004**			**2005**		
		**dry season**	**rainy season**	**global**	**dry season**	**rainy season**	**global**
**Data**	**Mean**	0.390	0.311	0.351	0.166	0.212	0.190
	**SD**^&^	0.385	0.260	0.330	0.241	0.257	0.250
**Kriging model**		Ordinary	Ordinary	Ordinary	Ordinary	Universal	Ordinary
**Transform data**		No	No	No	No	No	No
**Trend Removal**		No	No	No	No	Yes	No
**Searching Neighborhood**	**Radius (m)**	9924.7	9509.9	9509.9	9924.7	9509.9	9924.7
	**Angle**	0	0	0	0	0	0
**Prediction error measurements**	**Mean**	0.021	0.005	0.012	0.005	0	-0.0004
	**MSE**^#^	0.435	0.302	0.338	0.157	0.113	0.102
	**Average Stand. Error**	0.4	0.282	0.308	0.144	0.11	0.102
	**SMSE**^$^	1.09	1.074	1.098	1.088	1.015	1.006

^&^sd -standard deviation;^#^MSE-Mean Square Error;^$^SMSE- standardised mean square error;

**Figure 3:**
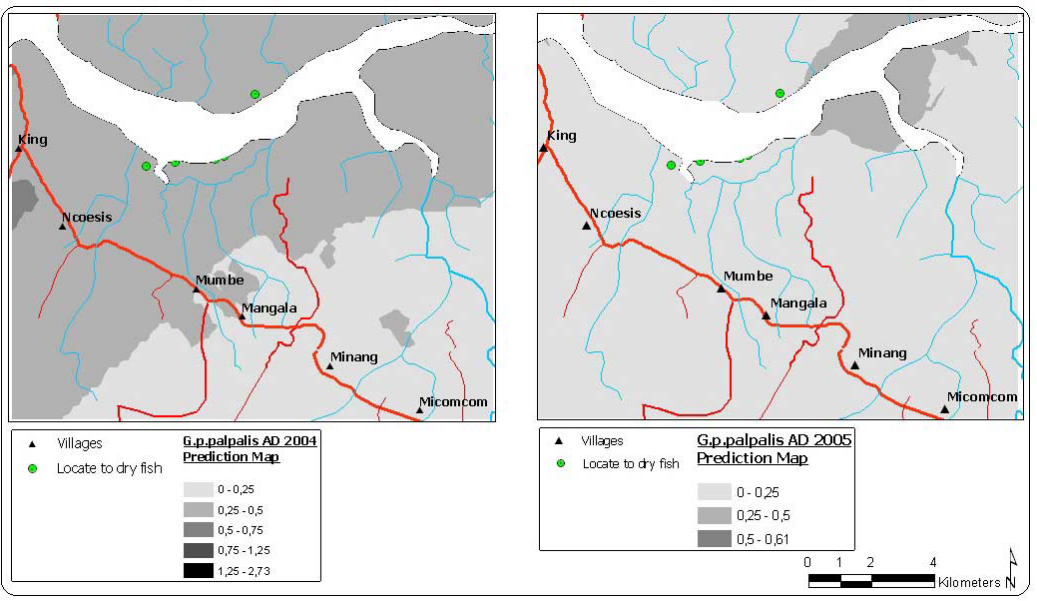
Prediction maps of the *G. p. palpalis *apparent density for 2004 and 2005.

**Figure 4:**
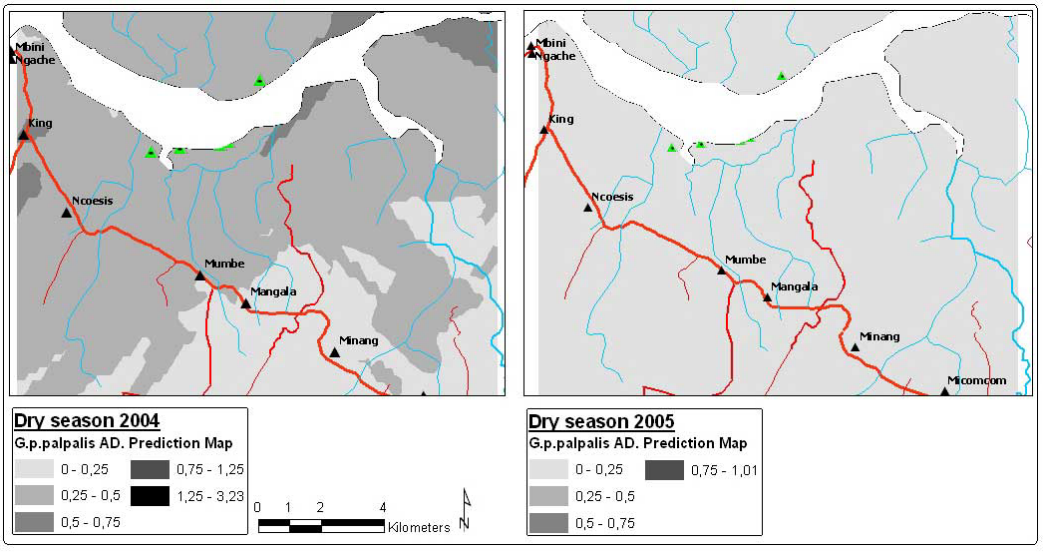
Prediction maps of the *G. p. palpalis *apparent density in dry season for 2004 and 2005.

**Figure 5:**
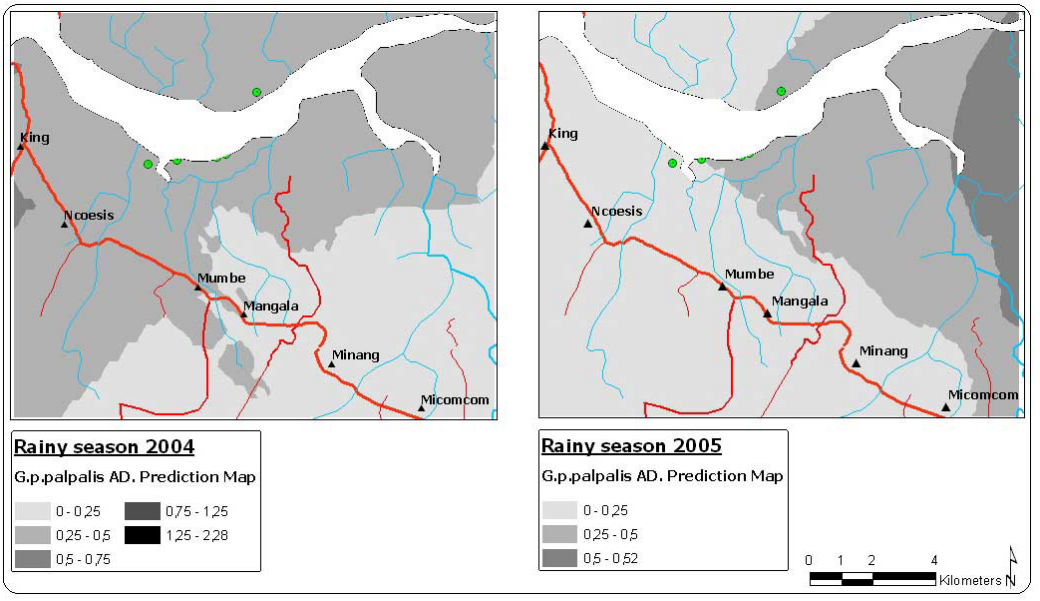
Prediction maps of the *G. p. palpalis *apparent density in rainy season for 2004 and 2005.

*G. p. palpalis *MAD in the Mbini focus is low and the distribution homogeneous, probably consequence of the outcome of the vector control carried out since 2002 (Figures 3 to 5). The 2004 prediction model reveals a zone where the density of *G. p. palpalis *is much higher (MAD: 0.25 to 0.5), a zone close to the estuary of the River Wele and tributaries. The density of this vector species decreases as the distance increases from the main waterway. In 2005, after one control year using mono-pyramidal traps, the *G. p. palpalis *population dropped notably throughout the focus. MAD values of between 0.25 and 0.5 were only maintained in the upper reaches of the river (Figure 3).

If the model generated for the seasonal AD is analysed, differences can be seen in the distribution of the *G. p. palpalis *population densities between the rainy and dry season. During the 2004 dry season, the highest *G. p. palpalis *densities (DAP > 0.5) were noted at some points of the focus epicentre (between King and Ncoesis locations) and the upper reaches of the River Wele. During the rainy season, a similar distribution pattern to the dry reason was noted, even though the MAD fell significantly in the aforementioned zones.

The lowest *G. p. palpalis *distribution was recorded in the 2005 dry season (DAP < 0.5), with a completely homogeneous distribution (Figure 4). During the rainy season of that same year, a recovery in the vector populations was noted, particularly in the upper reaches of the River Wele and tributaries, even though densities under 0.5 *G. p. palpalis*/trap/day continued to be recorded in the focus epicentre (Figure 5).

The difference between the 2004 and 2005 AD prediction surfaces provides an estimate of the impact of the mono-pyramidal traps on the man-vector contact. Thus, for the whole interpolation area, from one year to another, the *G. p. palpalis *AD has fallen by an average of 0.13 (sd: 0.06).

## Discussion

The apparent density of *G. p. palpalis *in the Mbini focus during the study period was low as a result of the control activity that had been carried out since 2002. During the first months of the study, the number of catches dropped sharply, even though at the end of the second year, a progressive recovery of the *G. p. palpalis *populations was noted (Figure 1). The increase in the number of disappeared, broken or fallen traps, mainly in villages where there was greatest resistance among the population towards the control activities, could be one of the reasons for this recovery. The sharp drop in the number of operative traps (from 0.15 to 0.10 traps per km^2^) took place halfway through the second year of study and was attributed to the immigration of citizens that arrived from non-endemic areas, who were not highly aware of the disease and control strategies. Therefore, it is essential to raise awareness among Mbini inhabitants and involve the communities in the vector control activities, which must always be supervised and checked by qualified workers [21].

On the other hand, as Figure 5 shows, the recovery began in the upper reaches of the River Wele, considered to be a low transmission risk due to the lack of human settlements. The percentage of mono-pyramidal traps located in the zone was low (30.5%), compared to those to be found at the sites in the focus epicentre.

In general, the trap density is considered to be determined by the abundance of tsetse flies and by the dispersion capacity of the species to be found in the zone [21]. Nash (1953) noted that the *G. p. palpalis *populations may travel up to 3 km from their breeding grounds [22] and that during the rainy period the dispersion of the populations is frequent along the waterways [23]. This behaviour would allow the spreading and re-colonization from untreated zones or with less pressure (lower trap density), aspects that were not taken into account when introducing the mono-pyramidal traps in Mbini. This has likely contributed to the fish dryers and jetties being the areas with the highest *G. p. palpalis *densities, even after the traps had been operative for a year. These zones are characterised for the predominance of the mangrove (*Rhizophora mangle, Avicennia nitida*) and riverbank forest, an ideal habit for this trypanosomiasis vector in Central Africa [24].

Emigration towards unpopulated areas implies a series of changes to the ecosystems that can foster the movement (withdrawal) of the tsetse fly populations [9]. Morris (1951) noted that a habitat reduction of the tsetse fly, particularly due to the elimination of the riverbank vegetation, can lead to a sustained reduction of the risk of infection [25]. However, the population movements, such as those arising from an economic activity, become a risk factor when they involve people entering into the natural habitat of the vector [11,14]. During the studies carried out in the Luba insular focus, Equatorial Guinea, a great incidence of cases was noted in the adult population that worked in cocoa plantations, the *G. p. palpalis *habitat on the island [15]. Therefore, despite the tsetse fly density being relatively low in fish dryers and jetties, the population that works in these zones (mainly fishermen) could be more exposed to infection.

The application of data interpolation and extrapolation techniques for the study of the spatial distribution of vector species should be performed with caution. The inappropriate use of these techniques may sometimes lead to more information, which is erroneous in many cases, being generated, than that provided by the data itself [2]. The prediction, or probabilistic, models obtained using these techniques represent the change of referenced spatially point observations, on a continuous surface area where the variable of interest is estimated for non-sampled location [26]. In order to apply these interpolation techniques (fundamentally *kriging*), the spatial structure of the data must be analysed and characterised beforehand [7].

The distribution of the traps was not random and was not carried out according to uniformity and spatial representativeness criteria. The uniform distribution of the sampling sites results in better data spatial variability being gathered and notably improves the quality of the estimates [7]. Therefore, the interpolation error will be greater the further the distance from the zones where the sampling is concentrated. In addition, the maximum autocorrelation distance of the data should be taken into account to establish the interpolation distance. These factors must be taken into account when interpreting the prediction maps. Therefore, the use of traps to establish and monitor the spatial pattern of vector distribution may require different distribution criteria than those used to control the vector populations, particularly when spatial analysis tools, such as the kriging, are going to be applied.

Despite the above, as can been seen in Table 3, the quality of the prediction surfaces of the annual and seasonal AD for *G. p. palpalis *is, in general, good, particularly for the second year of study.

## Conclusion

The mono-pyramidal traps in the Mbini focus have been proven to be a useful tool to control *G. p. palpalis*, even though the activity on the banks of the River Wele, including the unpopulated zones, needs to be intensified. Thus, the potential risk of infection for the temporary population in the zone would be reduced and recolonizations in the controlled areas would be avoided.

The application of spatial analysis techniques and the development of a geographical information system have allowed to study the distribution and dynamics of the *G. p. palpalis *populations in the Mbini focus, and they have been proven to be useful tools to discriminate zones with high and low AD of HAT vector, probably associated with different potential risk of sleeping sickness transmission.

## Methods

### Study area

**Figure 6:**
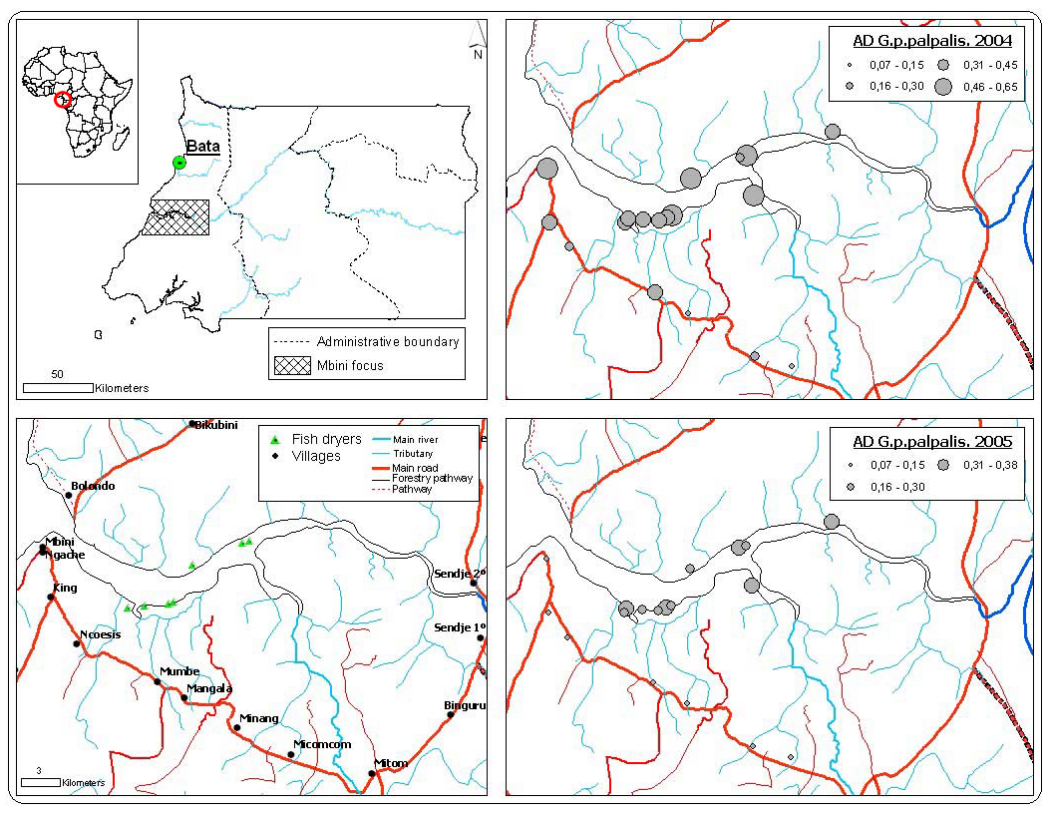
Distribution of pyramidal traps in the Mbini focus and mean apparent density for *G. p. palpalis *in each treatment zone (2004–2005).

Mbini is just a few kilometres from the capital of the continental region, Bata. In the Mbini district, there are 11,292 inhabitants, out of which 53% (5,939) are women. It is estimated that 83% (9,372) of the inhabitants live in rural areas. The population of Mbini lives off smallholdings (subsistence farming), artisanal fisheries and forestry. The fishing is fundamentally off the coast, even though fishing also takes place on the River Wele, the main waterway (Figure 6). The country's mainland region has a typically equatorial climate. Two dry seasons (December-March, June-September) and two rainy seasons (March-June, September-December) alternate. The annual rainfall amounts to 2074 mm over 118 rainy days. The annual relative humidity ranges between 70% and 100%. The average temperature is 25°C, with a minimum of 17°C and a maximum of 30°C. Local data about rainfall, average temperature and relative humidity per seasons and years is unavaliable in Equatorial Guinea.

### Tsetse flies collection and processing

In June 2004, 72 mono-pyramidal traps supplied by Veestergaard-Frandsen^®^, were placed in zones considered to have a high transmission risk, water points in sites where cases of trypanosomiasis has been diagnosed over the last 5 years. Taking into account that the study area has a total surface of 473.04 km^2^, the density of traps was 0.15 traps per km^2^. From the beginning of the control activities (2002), the same number of traps and locations point has been maintained.

The mono-pyramidal trap [27] was used, which as it includes a system to collect and conserve the tsetse flies (collection bottle with 5% Formalin solution), means it can be used to determine and monitor the entomological parameters, such as the apparent density. Furthermore, this type of traps has proven to be highly effective in controlling the tsetse fly populations [15,27,28]. The traps were checked every two weeks during the two first months of activity and monthly after the third. In the field, the tsetse flies were counted and preserved in absolute ethanol. Subsequently, the Brunhes J et al (1998) key was used to determine the species in the reference laboratory [29].

### Statistical Analysis

The apparent density per trap (AD), number of flies caught per trap and day, was established as the base and monitoring indicator in order to determine the effectiveness of the pyramidal traps in controlling the tsetse fly populations.

Furthermore, the apparent density of *G. p. palpalis *was calculated for each habitat identified as a high contact risk zone: pond-pools (7 traps), wells (10), type I jetties (in secondary tributaries) (4), small rivers (29), type II jetties (on River Wele) (9), and fish dryers (13).

Using non-parametric tests (Mann-Whitney test, Kruskal-Wallis test), the variability of the AD for each *glossina *species was analysed in terms of years and climatic seasons, and the AD of *G. p. palpalis *according to biotopes was studied. In all cases, values of p < 0.05 were considered to be statistically significant.

### Spatial analysis

The geographical location (longitude and latitude) of all the pyramidal traps was recorded using a GPS receiver. Using this information and the maps for the study zone provided by the INDEPROF (Institute of Forestry Promotion and Development, Equatorial Guinea), a GIS was developed for the spatial analysis of the studied entomological parameters.

In order to generate prediction surfaces, based on the information collected in detailed sampling, spatial interpolation methods, geostatistical or deterministic, were used. Kriging is a geostatistical method that includes a covariance model of the random function and uses the interpolation of a weighted moving average to generate the optimal spatial linear prediction [30],

Z(s) = μ(s) + ε(s),

where Z (s) would be the variable of interest that is composed by a deterministic trend μ(s) and an autocorrelated random error ε(s). The symbol "s" refers to a location (x, y) in space. The basic kriging approach is to consider the estimate of Z(s) as a linear combination of the available observations.

The *ordinary kriging *does not take into account the trend, and considers it to be constant and unknown throughout the model (μ(s) = m), while the trend in the universal kriging can be adjusted to a polynomial function.

The *ordinary kriging *and the *universal kriging *were used to estimated the annual and seasonal AD of *G. p. palpalis *throughout the epicentre of the Mbini focus for 2004 and 2005. The possible trends in the spatial distribution of the data were analysed and the directionality (anisotropy) in the spatial autocorrelation of the collected AD values was studied, and were included in the model when it was considered that they improved the prediction. The analysis was conducted using the *Geostatistical Analyst *extension included in the ArcGIS 9.1 software.

### Validation of the prediction models

In order to determine the reliability of the estimates provided by the prediction surface areas, a crossed validation method was used. This method consists in carrying out as many interpolations and measurements as in the model. In each process, the measurement value of one of the traps was produced and using a linear regression analysis, the correlation that exists between the mean value (observed) was compared to the prediction generated for that point with the rest.

As a result of this crossed validation, a series of parameters was obtained that determined the quality of the prediction: the mean and the mean standard prediction error, the mean square error (MSE) and the standardised mean square error (SMSE). In order for a model to be considered as providing a good prediction, the mean prediction error must be near to 0, the MSE and mean standard prediction error as small as possible, and the SMSE near to 1.

## Authors' contributions

JC was involved in the design of the survey, performed the GIS model and spatial analysis, participated in the collection of tsetse flies and the interpretation of statistical analysis, and coordinated the draft of the manuscript. MAD performed the statistical analysis and drafted the manuscript. PNA was involved in designing the study and approved the version to be published. NNM, SNO, LB, JNB, MOE participated in the collection and identifications of tsetse flies. AB and JR participated in the design of the study and approved the version to be published. All authors read and approved the final manuscript.
